# Melatonin to prevent delirium in patients with advanced cancer: a double blind, parallel, randomized, controlled, feasibility trial

**DOI:** 10.1186/s12904-020-00669-z

**Published:** 2020-10-21

**Authors:** Peter G. Lawlor, Marie T. McNamara-Kilian, Alistair R. MacDonald, Franco Momoli, Sallyanne Tierney, Nathalie Lacaze-Masmonteil, Monidipa Dasgupta, Meera Agar, Jose L. Pereira, David C. Currow, Shirley H. Bush

**Affiliations:** 1 Division of Palliative Care, Department of Medicine, University of Ottawa, 43 Bruyère Street, Ottawa, ON K1N 5C8 Canada; 2 Bruyère Research Institute, Ottawa, Canada; 3 Ottawa Hospital Research Institute, Ottawa, Canada; 4 Bruyère Continuing Care, Ottawa, Canada; 5 School of Epidemiology and Public Health, University of Ottawa, London, Canada; 6 Department of Geriatric Medicine, Department of Medicine, University of Western Ontario, London, Canada; 7 Centre of Cardiovascular and Chronic Care, Faculty of Health, University of Technology Sydney, Hamilton, Canada; 8 Division of Palliative Care, Department of Family Medicine, McMaster University, Hamilton, Canada

**Keywords:** Melatonin, Delirium, Advanced cancer, Feasibility study, Randomized controlled trial, Sleep, Pilot, Prevention

## Abstract

**Background:**

Delirium is highly problematic in palliative care (PC). Preliminary data indicate a potential role for melatonin to prevent delirium, but no randomized controlled trials (RCTs) are reported in PC.

**Methods:**

Patients aged ≥18 years, with advanced cancer, admitted to an inpatient Palliative Care Unit (PCU), having a Palliative Performance Scale rating ≥ 30%, and for whom consent was obtained, were included in the study. Patients with delirium on admission were excluded. The main study objectives were to assess the feasibility issues of conducting a double-blind RCT of exogenous melatonin to prevent delirium in PC: recruitment, retention, procedural acceptability, appropriateness of outcome measures, and preliminary efficacy and safety data. Study participants were randomized in a double-blind, parallel designed study to receive daily melatonin 3 mg or placebo orally at 21:00 over 28 days or less if incident delirium, death, discharge or withdrawal occurred earlier. Delirium was diagnosed using the Confusion Assessment Method. Efficacy endpoints in the melatonin and placebo groups were compared using time-to-event analysis: days from study entry to onset of incident delirium.

**Results:**

Over 16 months, 60/616 (9.7%; 95% CI: 7.5–12.4%) screened subjects were enrolled. The respective melatonin (*n* = 30) vs placebo (*n* = 30) outcomes were: incident delirium in 11/30 (36.7%; 95%CI: 19.9–56.1%) vs 10/30 (33%; 95% CI: 17.3–52.8%); early discharge (6 vs 5); withdrawal (6 vs 3); death (0 vs 1); and 7 (23%) vs 11 (37%) reached the 28-day end point. The 25th percentile time-to-event were 9 and 18 days (log rank, χ^2^ = 0.62, *p* = 0.43) in melatonin and placebo groups, respectively. No serious trial medication-related adverse effects occurred and the core study procedures were acceptable. Compared to those who remained delirium-free during their study participation, those who developed delirium (*n* = 21) had poorer functional (*p* = 0.036) and cognitive performance (*p* = 0.013), and in particular, poorer attentional capacity (*p* = 0.003) at study entry.

**Conclusions:**

A larger double-blind RCT is feasible, but both subject accrual and withdrawal rates signal a need for multisite collaboration. The apparent trend for shorter time to incident delirium in the melatonin group bodes for careful monitoring in a larger trial.

**Trial registration:**

Registered on July 21st 2014 with ClinicalTrials.gov: NCT02200172.

## Background

Delirium is a recognized complication of medical illness, particularly in older patients, in whom it is associated with increased mortality, hospitalization and healthcare costs [1]. In inpatient palliative care settings, the prevalence of delirium has been reported in the 20–42% range on admission and up to 88% in the last hours or days of life [2]. Delirium is an acute neurocognitive disturbance in which one’s awareness of the immediate environment is decreased and disordered attentional capacity is a core feature; other cognitive deficits and perceptual abnormalities may also occur [3]. As a consequence of these features and associated hypo- or hyperactive psychomotor changes, normal communication is impeded. The collective syndromal features of delirium invariably generate distress for patients and their families [4]; for healthcare practitioners, delirium presents a barrier in symptom assessment and a clinical management challenge, particularly when psychomotor agitation occurs [5–7]. This is especially true in the context of advanced disease and end-of-life care, in which patients’ physical and functional decline confers a high level of baseline vulnerability towards delirium precipitants such as infection and adverse medication effects [6].

The clinical management approach towards an episode of delirium in the palliative care context is to identify and treat correctable precipitating factors, if consistent with the patient’s desired goals of care; in situations where the desired goals of care are solely focussed on comfort, or where the delirium precipitants are refractory to treatment, therapeutic intervention must then focus on symptomatic management of distressing symptoms such as perceptual disturbance or agitation [8]. Antipsychotics have been advocated in the first line pharmacological management of distressing delirium symptoms [9, 10]. However, evidence is emerging that antipsychotics have no preventative role and a limited therapeutic role for delirium in hospitalized adults [11–13]; in a recent trial in palliative care patients, antipsychotics were in fact associated with worsening of mild to moderate delirium when compared to placebo [14]. The recommended overall management approach is shifting towards greater preventive efforts, especially with non-pharmacological interventions and minimizing antipsychotic use where possible [15]. Multi-component non-pharmacological interventions, including maintenance of sleep hygiene have remarkable efficacy in preventing delirium in older people, [16, 17] but similar interventions together with deprescribing showed no benefit in preventing delirium in a palliative care population study [18]. Sleep-wake cycle disturbance is not a core diagnostic criterion for diagnosis of delirium, but its prevalence in cancer patients with delirium has been reported in the 75–100% range [19, 20]. Although the pathophysiology of delirium is complex and not fully understood, melatonin dysregulation and associated sleep-wake cycle disturbance is postulated as one of the mechanisms in the pathogenesis of delirium [21].

Melatonin is a natural hormone that is secreted predominantly by the pineal gland, particularly in response to darkness onset, coinciding with the initiation and maintenance of sleep; it has a major role in both the regulation and synchronization of the sleep-wake cycle and circadian rhythms [22]. In addition to its chronobiotic role, melatonin has diverse and complex oncostatic and immunomodulatory properties [23–25]. Furthermore, altered circadian melatonin levels, particularly a reduction in peak endogenous production, have been reported in relation to various cancers, [26–28] healthy ageing and cognitive impairment [29, 30]. Disturbed circadian production of melatonin has also been reported in postoperative patients and those with critical illness, particularly sepsis [31–33]. Melatonin dysregulation has thus been demonstrated in most of the clinical populations at highest risk of delirium, which is the main hypothetical basis of exogenously administering melatonin to prevent delirium.

Although there is growing interest in the role of melatonin and melatonin receptor agonists in promoting sleep and preventing delirium in critical care and other high-risk populations, [34] heterogeneity among delirium prevention studies with melatonin supplementation precludes broad conclusive recommendations [35]. Although two systematic reviews and meta-analyses failed to show statistically significant benefit in delirium prevention for melatonin or its agonist, ramelteon across postoperative, intensive care and older medical populations, [36, 37] a sub-group analysis estimated that melatonin administration decreased the incidence of delirium by 75% in older (> 65 years) medical patients [36]. Meanwhile, delirium prevention studies with melatonin supplementation have not been published to date in the palliative care population, whose clinical characteristics are likely to have a shared overlap with the older medical population.

Given the rationale for a randomized controlled trial (RCT) to evaluate the role of melatonin in preventing delirium in the palliative care population, and prior to conducting such a trial, hereafter referred to as the main study, a preliminary study was conducted to examine its feasibility. The primary objectives of this feasibility study were to determine (1) estimates of participant recruitment and retention, (2) the appropriateness of the main study’s outcome measures, based on both the frequency of protocol violation and ascertainment of preliminary data on time to onset of first incident episode of delirium, cumulative incidence of delirium, incident rate of delirium and the severity of delirium and insomnia (3) the acceptability of study procedures, including assessment tools to patients, their families and palliative care unit staff. The secondary objectives of this feasibility study were to (a) assess the feasibility of data collection regarding predisposing and precipitating risk factors for delirium, (b) facilitate the initial implementation of standard delirium prevention and management guidelines on the palliative care unit, and (c) assess the safety of the study interventions, based on preliminary estimates of adverse events.

## Methods

The methods have been described in detail in a previous protocol publication and will be summarized here [38].

### Study setting, design and sample size

The feasibility study was conducted in a 31-bed inpatient, university teaching palliative care unit (PCU) at Élisabeth Bruyère Hospital, Ottawa, Canada. The study was a double-blind, randomized, parallel arm, placebo-controlled, single centre trial of once daily, orally administered melatonin to prevent delirium in patients with advanced cancer. The proposed primary outcome of the main study is time-to-event survival (survival in this context refers not to life survival but to remaining delirium-free, albeit at risk of delirium) analysis for first incident episode of delirium. This means that participants who do not develop delirium by the end of the study period would be censored in the analysis; similarly, those participants who might leave the study without developing delirium before the designated study period ends would contribute at-risk time to the time-to-event survival analysis, but would be censored at the time of their leaving the study.

For time-to-event survival analysis, we anticipated a 25% incidence of delirium in the placebo arm of the main study. Accounting for censoring, we estimated a sample size requirement of *N* = 410 to detect an effect size (hazard ratio) of 0.5, given an alpha level of 0.05 and power of 80% in the main study. Consistent with literature recommendations, [39] a sample size requirement of 60 (30 in each arm) was estimated for the feasibility study, which was 15% of the sample size (N = 410) of the main study. Feasibility targets were not formally pre-specified for this single site study in anticipation that the main study would likely require multisite participation.

The feasibility study assessed the proposed primary and secondary outcomes of the main study: time in days from study enrollment to first inpatient episode of delirium (event) for 50% (often referred to as median survival time) of each study group in the at-risk population; cumulative incidence of delirium within the study period of 28 days; incidence rate of delirium per person-time, also known as incidence rate density; severity of incident delirium and perceived sleep satisfaction. The use of person-time incidence and time-to-event survival outcomes rather than cumulative incidence within a certain time interval accounts for those situations where the observation time differs between participants, or the population at risk varies with time.

### Study eligibility

Inclusion criteria: age ≥ 18 years; documented diagnosis of advanced cancer; admitted to the inpatient PCU; a rating ≥ 30% on the Palliative Performance Scale (PPS) [40]; cognitive capacity to give informed consent, as assessed by the attending physician, or if cognitively impaired, access of the study team to a substitute decision maker (SDM) to obtain informed written consent.

Exclusion criteria: delirium present on admission, based on a positive CAM assessment; known psychotic disorder other than dementia; inability to take medications sublingually or via gastrostomy tube; known allergy to melatonin or placebo content; use of melatonin within the 2 weeks preceding admission; patients on warfarin or other oral anticoagulants; on other investigational agents or treatments or on immunosuppressant medication in the context of autoimmune disease or post organ transplantation; communication problems that could not be accommodated in the course of study assessments, including deafness, tracheostomy, aphasia, dysarthria or emotional distress; severe visual impairment or designated legally blind; and pregnancy or lactation.

### Recruitment and randomization

Consecutive potential study participants were approached within 72 h of admission and assessed by their attending physician regarding study eligibility criteria; those eligible were referred to either a trained clinical research nurse (CRN) or trained clinical research assistant (CRA) from the study team, who provided information verbally and in writing, and obtained informed written consent before study enrollment of all participants.

Using the Ottawa Hospital Research Institute (OHRI) Data Management Services (a web-based randomization system), a central randomization master list, incorporating a 1:1 ratio for melatonin to placebo, was pre-generated by an independent statistician and sent by secure electronic communication to the hospital’s Director of Pharmacy. The melatonin drug and identical placebo were packaged, and numbered as per the master list. This list was kept confidential and secure by OHRI Data Management Services, the Director of Pharmacy and the specific pharmacy technician who prepackaged the study (melatonin or placebo) medication. The label of the study medications only broadly identified the contents as “Melatonin/ Placebo Study Drug”. Allocation of study group (melatonin or placebo) was concealed: the study investigators, research nurse and assistant, the PCU pharmacist, nurses, physicians, dispensing pharmacist and technician, and study participants were blinded to the exact study medication.

### Study drug administration protocol and safety monitoring

Enrolled subjects were randomised to receive melatonin 3 mg (immediate release) or an identical (size, shape and taste) placebo at 21:00 (+/− 1 h) on study day 1 (D1) and daily thereafter until D28 or earlier in the event of death, discharge, study withdrawal or diagnosis of incident delirium. The study drugs were supplied by Jamieson Laboratories Ltd., Windsor, Ontario, Canada.

The National Cancer Institute’s Common Terminology Criteria for Adverse Events (NCI-CTAE, v 4.03) format was used for adverse event recording and reporting [41]. An independent Data and Safety Monitoring Board (DSMB) was established for the study and received regular standardized reports from the study team.

### Additional measures and data recording

Routine physician assessments on the day of PCU admission include the Confusion Assessment Method (CAM), [42, 43] a validated tool to screen for and diagnose delirium, and the Short Orientation Memory Concentration Test (SOMCT), [44] a validated tool to assess cognition. Routine nursing assessments included 8-hly delirium screening on a daily basis with the Nursing Delirium Screening Scale (Nu-DESC), a validated observational tool (score range 0–10) that was completed in relation to each 8-hour nursing shift [45]. A score ≥ 2 on the Nu-DESC prompted an attending physician CAM rating within 24 h. Symptom intensity is routinely rated using the revised Edmonton Symptom Assessment System (ESAS-r) [46] and cancer pain is classified using the Edmonton Classification System for Cancer Pain (ECS-CP) [47].

Specific study assessments included the following: the Insomnia Severity Index (ISI), [48] which was conducted on D1 (Study Day 1), D14 ± 2 days and D28 ± 2 days; the Memorial Delirium Assessment Scale (MDAS), [49] which was rated on weekdays (with reference to the preceding 24 h) by the CRN within 24 ± 8 h of incident delirium diagnosis; and the Clinician Global Rating (CGR) of delirium severity (mild, moderate or severe), which was rated by the attending physician at the time of incident delirium diagnosis.

The designated goals of care, based on consensual input from patient and/or SDM, were recorded at admission and at the day of diagnosis of incident delirium. Consistent with patient and family wishes, the designated goals of care help to determine the degree of intensity applied to the investigation and treatment of an episode of delirium. This approach is consistent with the Canadian Guidelines on the Assessment and Treatment of Delirium in Older Adults at the End of Life, [50] which were adapted and introduced at the commencement of this feasibility study. The goals of care categories were as follows: “R” for full resuscitation; “M + T” for medical and transfer (indicating full medical investigation and treatment on the PCU with the option to transfer out to tertiary acute care as necessary for additional investigations or treatments); “M” for medical investigation and treatment without transfer out; and “C” for comfort only care.

Data relating to baseline or predisposing factors (at D1) and acute onset precipitating factors at the time of incident delirium were collected by the CRN or CRA using standard checklists (see Additional files 1 and 2, Table S1 and Table S2 with checklists for baseline/predisposing factors and acute onset precipitating factors, respectively). Within 3 days of delirium diagnosis, the CRN or CRA also interviewed the attending physician of those study patients who developed incident delirium and systematically recorded a checklist of potential precipitant risk factors (categorized as definite, probable, possible, present but apparently not contributory, and either ruled out or not present or not relevant) associated with the episode of incident delirium. Laboratory and other investigations were conducted as per routine practice on the PCU and consistent with the designated goals of care. Laboratory abnormalities were designated as such based on their being outside of the local laboratory reference range in all cases. The total opioid dose in morphine oral equivalent, the corticosteroid daily dose in prednisone oral equivalent, the diazepam oral equivalent benzodiazepine daily dose and the total anticholinergic drug scale (ADS) score at D1 were calculated in accordance with standard tables and references [51–53]. The Charlson Co-morbidity Index was rated on D1 [54].

### Data analysis

The data analysis for the feasibility study was largely descriptive. Categorical data were summarized as proportions or percentages with 95% confidence intervals (CIs), unless otherwise stated; continuous data were summarized as medians with interquartile (Q1-Q3) ranges, as most were not normally distributed. As a preliminary report, statistically significant differences between the study groups were analyzed using the Pearson Chi Square or Fisher’s Exact Tests for categorical variables and the Mann-Whitney Test for continuous variables. Similarly, preliminary data regarding the time to event of the first episode of delirium in the two study groups was compared using a Kaplan-Meier analysis. Statistical analyses reported in this paper were conducted using Stata 14.1 (StataCorp LP, College Station, TX, USA).

### Ethical approval and oversight requirements

A no objection letter was obtained from the Therapeutic Products Directorate of Health Canada to proceed with the study, which was sponsored by Bruyère Research Institute. The study received ethical approval from the Ottawa Health Sciences Network and Bruyère Research Ethics Boards. In addition to providing regular reports to the DSMB, an independent study monitor, who conducted a site visit and review of procedures at the times of 20th, 40th and 60th subject recruitment, was also appointed. The trial was registered on July 21st 2014 with ClinicalTrials.gov: NCT02200172. The study adheres to CONSORT guidelines for randomized pilot and feasibility trials [55].

## Results

### Recruitment, retention and demographic characteristics of participants

**Fig. 1:**
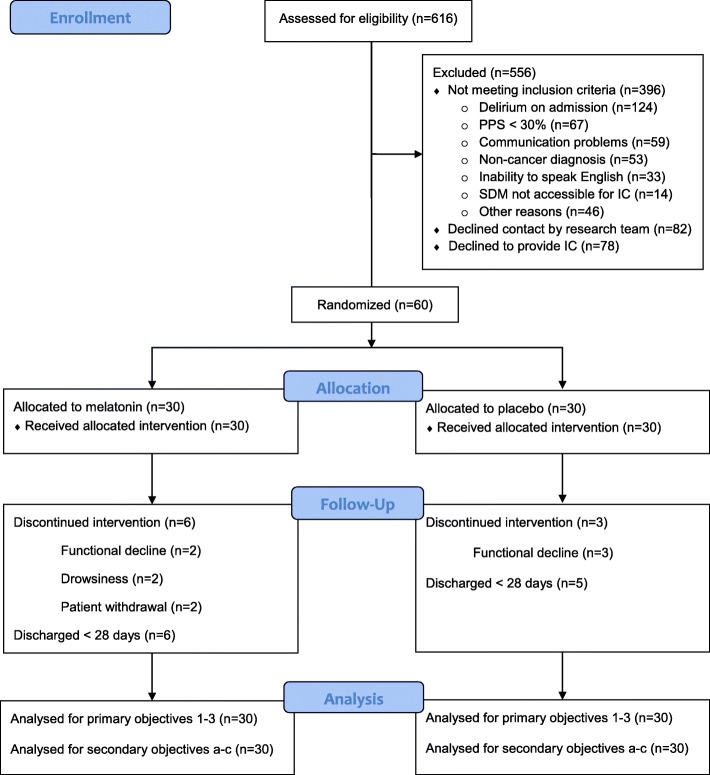
CONSORT Flow Diagram. PPS: Palliative Performance Scale; SDM: Substitute Decision Maker; IC: Informed Consent

Over the course of 16 months from December 2014 to March 2016, 60/616 (9.7%; 7.5–12.4%) screened subjects were enrolled, 30 to each study arm. The numbers of excluded and included study subjects, and those in each arm with study end points reached prior to 28 days, due to discontinuation of study drug or study withdrawal, death or discharge are summarized Fig. 1**.**

Of the 556 excluded patients, 396 failed to meet the study eligibility requirements, most commonly due to delirium on admission in 124/396 (31%), low PPS rating (*n* = 67, 17%) and communication problems in (*n* = 59, 15%). Seven (23.3%; 9.9–42.3%) of the melatonin and 11 (36.7%; 19.9–56.1%) of the placebo study groups, a total of 18 (30%; 18.9–43.2%) completed the study to its designated full duration of 28 days without developing incident delirium. Among the remaining 21 (35%; 23.1–48.4%) participants whose study participation ended before 28 days without incident delirium being recorded, 1 from the placebo group died; 6 versus 3 were withdrawn and 6 versus 5 were discharged in the melatonin and placebo groups, respectively. The study exit for all of these participants was within the first 21 days.

The demographic, oncologic and comorbidity characteristics of the 60 study participants are summarized according to the randomized study groups in Table 1. The median age (interquartile range) of the entire study sample was 67 (60–75) years, with an almost identical distribution across the two study groups. Twenty-seven (45%) of the study sample were female, with a similar sex distribution across the two study groups. The most common cancer in the study sample was lung cancer, comprising of 20 (33%) of all of the cancers. The distribution of both primary cancer and metastatic sites was similar across the two study groups. One third of the study sample had metastatic brain disease and 10% had leptomeningeal disease. Median PPS and CCI values were 40 (30–50) and 10 (9–12), respectfully. Of the 60 study participants, 37 (61.7%) had their goals of care designated as medical at admission, whereas at the time of diagnosis of incident delirium, 8 (38%) and 12 (57.1%) of 21 participants with delirium had their goals of care designated as medical and comfort only, respectively. There were no statistically significant differences between the two study groups in relation to any of the baseline demographic, oncologic or comorbidity characteristics, including cancer pain mechanism, presence of incident pain or ESAS-r symptom intensity profiles. ESAS-r scores were most often missing for anxiety and drowsiness: 5 ratings were missing for each of these in the placebo group.

**Table 1:** Demographic, baseline oncologic and comorbidity characteristics in randomized study groups

Characteristic	Study Group		All patients	***P*** value
	Placebo ***n*** = 30 (%)	Melatonin ***n*** = 30 (%)	Total ***n*** = 60 (%)	
**Age, yrs**^a^	67 (60–76)	67 (59–75)	67 (60–75)	0.531
**Sex: female**	13 (43.3)	14 (46.7)	27 (45.0)	0.795
**Primary cancer**				
**Lung**	12 (40.0)	8 (26.7)	20 (33.3)	0.531
**Gastrointestinal**	7 (23.3)	9 (30.0)	16 (26.7)	
**Genitourinary**	4 (13.3)	4 (13.3)	8 (13.3)	
**Breast**	4 (13.3)	2 (6.7)	6 (10.0)	
**Hematologic**	1 (3.3)	1 (3.3)	2 (3.3)	
**Head & Neck**	1 (3.3)	0 (0.0)	1 (1.67)	
**Primary Brain**	0 (0.0)	2 (6.7)	2 (3.3)	
**Other**	1 (3.3)	4 (13.3)	5 (8.3)	
**Metastatic sites of cancer**				
**Lung**	8 (26.7)	12 (40.0)	20 (33.3)	0.273
**Liver**	12 (40.0)	14 (46.7)	26 (43.3)	0.602
**Brain**	12 (40.0)	8 (26.7)	20 (33.3)	0.273
**Leptomeningeal**	5 (16.7)	1 (3.3)	6 (10.0)	0.195
**Bone**	17 (56.7)	14 (46.7)	31 (51.7)	0.438
**Palliative Performance Scale (PPS)**^a^	40 (30–50)	40 (30–50)	40 (30–50)	0.613
**Charlson Comorbidity Index (CCI)**^a^	10 (9–11)	10 (9–13)	10 (9–12)	0.270
**Baseline Goals of Care**				
**Comfort only**	6 (20.0)	7 (23.3)	13 (21.7)	0.750
**Medical**	19 (63.3)	18 (60.0)	37 (61.7)	
**Medical & Transfer**	5 (16.7)	4 (13.3)	9 (15.0)	
**Full Resuscitation**	0 (0.0)	1 (3.3)	1 (1.67)	
**ECS-CP**^**b**^ **Pain mechanism**				
**No pain syndrome**	4 (13.3)	8 (26.7)	12 (20.0)	0.076
**Nociceptive**	18 (60.0)	9 (30.0)	27 (45.0)	
**Neuropathic**	8 (26.7)	12 (40.0)	20 (33.3)	
**Unknown or missing**	0 (0)	1 (3.3)	1 (1.67)	
**ECS-CP**^**b**^ **Incident pain**				
**Present**	15 (50.0)	13 (43.3)	28 (46.7)	0.606
**Absent**	14 (46.7)	17 (56.7)	31 (51.7)	
**Unknown or missing**	1 (3.3)	0 (0)	1 (1.67)	
**ESAS-r symptom intensity,**^c^				
**Pain**^a^	3 (2–3)	2 (0–5)	2.5 (1–4)	0.786
**Tiredness**^a^	5 (3–6)	4.5 (2–6.5)	5 (3–6)	0.525
**Drowsiness**^a^	3 (1–6)	4.5 (2–6.5)	4 (1–6)	0.413
**Nausea**^a^	0 (0–2)	0 (0–0.5)	0 (0–1)	0.486
**Lack of appetite**^a^	3.5 (0–7)	3.5 (0–7)	3.5 (0–7)	0.923
**Short of breath**^a^	2 (0–4)	0 (0–2)	1 (0–3)	0.072
**Depression**^a^	1 (0–3)	0 (0–3)	0.5 (0–3)	0.584
**Anxiety**^a^	1 (0–4)	2 (0–5)	2 (0–4)	0.787
**Well being**^a^	4.5 (2–6)	3.5 (2–5.5)	4 (2–6)	0.644
**Sleep**^a^	4 (0–6)	3.5 (0–6)	4 (0–6)	0.847

^a^Continuous variables expressed as median (Q1-Q3)

^b^ECS-CP: Edmonton Classification System for Cancer Pain

^c^ESAS-r: Edmonton Symptom Assessment System Revised; number of missing ratings (placebo, melatonin) for each symptom: pain (4, 2); tired (4, 2); drowsiness (5, 2); nausea (4, 2); lack of appetite (4, 2); short of breath (4, 2); depression (4, 2) anxiety (5, 2); well being (4, 2); sleep (7, 2)

### Baseline neurocognitive assessment

The results of baseline neurocognitive status assessments in both study groups are summarized in Table 2**.** Although one third of participants had university or third level education, the educational status of 26 (43.3%) was unknown. Consent was obtained from a SDM for 9 (15%) study participants. The distribution of patients with a documented diagnosis of dementia or cognitive impairment without dementia was similar in both study groups, as was the SOCMT score, which had a median of 2 (0–6) for the entire study sample. The presence or absence of a documented episode of delirium in the last year preceding their admission to the palliative care unit was unknown in 11 (18.3%) participants; 13 (21.7%) participants had a documented previous episode in this time period. There were no statistical differences between study groups in relation to most neurocognitive parameter assessments: documented episodes of delirium; documented diagnoses of mood or anxiety disorders; and neurocognitive symptom profiles, as obtained through the admission history from the patient or SDM. The neurocognitive symptom profiles included sleep pattern disturbance, which was present in 10 (16.7%) participants, and the median ISI score was 9 (3–13) for the entire study sample. There were also no differences between the study groups regarding the number of psychoactive medications prescribed or their doses. The missing data in relation to baseline neurocognitive parameters were highest for diazepam equivalent dose: 6/30 (20%) participants in the melatonin group had missing information for this.

**Table 2:** Baseline neurocognitive status and psychoactive medication profiles of study groups

Characteristic	Study Group		All patients	***P*** value
	Placebo ***n*** = 30 (%)	Melatonin ***n*** = 30 (%)	Total ***n*** = 60 (%)	
**Highest educational level achieved**				
**Elementary**	0 (0.0)	2 (6.7)	2 (3.3)	0.369
**Junior High School**	2 (6.7)	1 (3.3)	3 (5.0)	
**High School**	4 (13.3)	5 (16.7)	9 (15.0)	
**University / 3rd level**	8 (26.7)	12 (40.0)	20 (33.3)	
**Unknown**	16 (53.3)	10 (33.3)	26 (43.3)	
**Consent obtained**				
**Patient**	24 (80.0)	26 (86.7)	50 (83.3)	0.731
**Substitute**	6 (20.0)	4 (13.3)	9 (15.0)	
**Dementia diagnosis documented**^a^	2 (6.7)	2 (6.7)	4 (6.7)	1.000
**Cognitive impairment without dementia**				
**Documented present**	2 (6.7)	2 (6.7)	4 (6.7)	0.748
**Unknown**	1 (3.3)	2 (6.7)	3 (5.0)	
**Short Orientation Memory Concentration Test Score (SOMCT)**^b^	2 (0–5)	3 (0–7)	2 (0–6)	0.307
**Previous delirium episode**				
**Documented record of occurrence**	7 (23.3)	6 (20.0)	13 (21.7)	0.521
**Unknown**	7 (23.3)	4 (13.3)	11 (18.3)	
**Mood disorder diagnosis documented**^a^	2 (6.7)	6 (20.0)	8 (13.3)	0.357
**Anxiety disorder documented**^a^	3 (10.0)	3 (10.0)	6 (10.0)	0.891
**Recent neurocognitive symptoms (per history from patient or substitute)**^c^				
**Perceptual disturbance**	3 (10.0)	3 (10.0)	6 (10.0)	0.665
**Delusional disturbance**	0 (0)	0 (0)	0 (0)	0.385
**Attention problems**^a^	5 (16.7)	7 (23.3)	12 (20.0)	0.658
**Sleep pattern disturbed**^a^	8 (26.7)	2 (6.7)	10 (16.7)	0.080
**Insomnia severity index (ISI) score**^b^	11 (4–15)	7.5 (2–13)	9 (3–13)	0.199
**Psychoactive medications at admission**				
**Number prescribed**^b^	3 (1–4)	2 (1–3)	2 (1–4)	0.125
**Morphine equivalent daily dose (MEDD)**^bd^	60 (6–90)	37.5 (4–240)	54 (4.5–132)	0.645
**Diazepam equivalent daily dose**^bd^	0 (0)	0 (0)	0 (0)	0.931
**Prednisone equivalent daily dose**^bd^	11 (0–40)	16.25 (0–45)	12.25 (0–40.0)	0.757

^a^Missing data in study groups (placebo, melatonin) for categorical variables: dementia diagnosis documented (0, 1); mood disorder diagnosis documented (1, 1); anxiety disorder documented (2, 4); attention problems (4, 2); sleep disturbed (0, 1); missing data were counted as values in statistical analyses

^b^Continuous variables expressed as median (Q1-Q3); missing data in study groups (placebo, melatonin) for continuous variables: SOMCT score (2, 3); ISI score (1, 0); psychoactive medications (1, 3); diazepam equivalent (4, 6); prednisone equivalent (0, 2)

^c^Subsyndromal delirium features in the absence of a documented diagnosis of delirium

^d^Oral equivalent in milligrams

### Preliminary data on the outcome measures chosen for main study

**Fig. 2:**
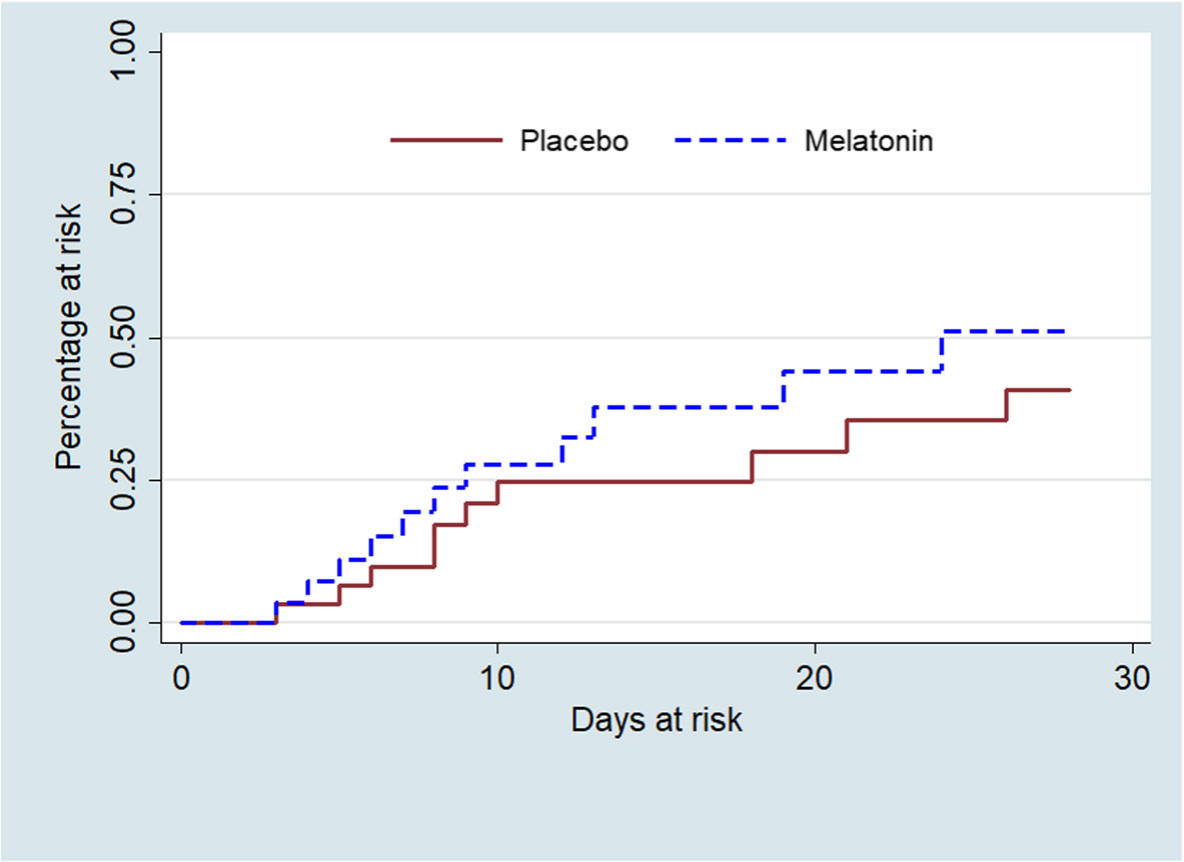
Kaplan-Meier plots of cumulative incidence of delirium in study groups

The assessment measures recorded in relation to incident delirium in the two study groups are compared in Table 3**.** Incident delirium occurred in 21 (35%; 23.1–48.4%) of participants; of these 10 (33.3%; 17.3–52.8%) were in the placebo group and 11 (36.7%; 19.9–56.1%) in the melatonin group. The incidence rate or incidence density of delirium was 0.019 or 1.9 cases/100 person-days in the placebo group and 0.027 or 2.7/100 person-days in the melatonin group. This resulted in an incidence rate ratio of 1.40 (95% CI: 0.595–3.27) for the melatonin compared to the placebo group (*p* = 0.45). As only the melatonin group had reached the 50th percentile of the at-risk study population by the end of the study period, the first quartile delirium-free survival was used instead of the median survival for comparison of the study groups. Thus, the time in days for the first quartile of the at-risk study population to have an event (delirium) was 9 and 18 days (log rank, χ^2^ = 0.62, *p* = 0.43) in the melatonin and placebo groups, respectively (see Fig. 2).

**Table 3:** Incident delirium assessment measures

Characteristic	Study Groups		All patients	***P*** value
	Placebo ***n*** = 30 (%)	Melatonin ***n*** = 30 (%)	Total ***n*** = 60 (%)	
**Incident Delirium Diagnosis (IDD)**				
**Cumulative 28-day incidence**	10 (33.3)	11 (36.7)	21 (35.0)	0.787
**Total person-days at risk**	521	411	932	
**Incidence rate per person-day [95% Confidence interval]**	0.019 [0.010–0.036]	0.027 [0.015–0.048]	0.023[0.015–0.036]	0.227
**First quartile delirium-free survival time (days)**	18	9	10	0.433
**Severity measures at IDD**				
**MDAS**^a^ **completed < 24 h of IDD**	6 (60.0)	6 (54.5)	12 (57.1)	0.331
**MDAS score**^**b**^	13.5 (11–17)	18 (16–20)	16.5 (12–18)	0.090
**Clinician Global Rating (CGR)**				
**Mild**	2/10 [20.0]	3/11 [27.3]	5/21 [23.8]	0.632
**Moderate**	6/10 [60.0]	8/11 [72.7]	14/21 [66.7]	
**Severe**	2/10 [20.0]	0/11 [0]	2/21 [9.5]	
**Nu-DESC ratings**^**c**^				
**Total number of ratings available over 72 h**^**d**^ **at IDD**^**b**^	7 (6–9)	8 (6–9)	8 (6–9)	0.462
**Maximum total Nu-DESC score over 72 h**^**d**^ **at IDD**^**b**^	4 (3–6)	3 (1–5)	3 (2–5)	0.191
**Maximum total Nu-DESC score during day of IDD**^**b**^	6 (4–7)	3 (3–5)	4 (3–6)	0.144

^a^*MDAS* Memorial Delirium Assessment Scale; in the placebo group, 4 MDAS ratings were missing, and in the melatonin group, 5 were missing

^b^Median (Q1-Q3)

^c^Nu-DESC: Nursing Delirium Screening Scale Eastern; score range 0–10 relating to 8-h nursing shift

^d^72-h period refers to calendar day prior to day of IDD, the day of IDD, and the calendar day following the day of IDD

In regard to the severity of incident delirium, there were 9 participants with missing MDAS ratings for their first 24 h of incident delirium; comparison of the six available MDAS ratings in each group revealed higher severity in the melatonin versus placebo group: 18 (16–20) versus 13.5 (11–17), (*p* = 0.09). There were no missing CGRs of delirium severity and rating categories (mild, moderate and severe) were not statistically different in the study groups. Similarly, there were no statistically significant study group differences in the maximum Nu-DESC score either during the day of incident delirium diagnosis or in the 72-h period that included the day before the day of incident delirium diagnosis, the day of incident delirium diagnosis and the day following this.

In rating the severity of insomnia, there were 28 (53%) of participants with missing D14 ISI ratings and 43 (72%) with missing D28 ISI ratings. Apart from a reported ISI baseline comparison, in which treatment group differences were not statistically significant, no further study group comparisons were made in view of the extent of missing data.

There was one protocol violation in the placebo group: two doses of the placebo medication were missed in one participant. There were 31 protocol deviations in the placebo group and 18 in the melatonin group, mostly relating to documentation and timing of assessments.

### Acceptability of study procedures

Field notes were made by the CRN and CRA regarding the acceptability of core study procedures. Apart from issues with assessment tools, the study procedures were otherwise considered acceptable. Missing assessments occurred most frequently in relation to the MDAS ratings and ISI ratings. Missing MDAS ratings were attributed to lack of weekend CRN/CRA assessor coverage in 3 participants and patient fatigue or distress in the remainder. In the case of participants with missing ISI ratings, many had either been discharged or become delirious before their ISI assessments that were scheduled on D14 or D28, or were noted to be too fatigued to complete the assessment.

### Secondary objectives

#### Data collection regarding delirium risk factors

The distribution of potential baseline risk factors in the 21 study participants who developed incident delirium is compared to that of the 39 participants who remained delirium free during the duration of their study participation is summarized in Table 4**.** Statistically significant differences were determined for 3 potential risk factors: compared to those who did not develop delirium, the participants who developed delirium had poorer functional status, as reflected by median (Q1-Q3) PPS scores of 40 (30–40) versus 40 (30–50), *p* = 0.036; higher SOMCT scores (reflecting poorer cognitive performance at baseline), 6 (2–10) versus 2 (0–4), *p* = 0.013; and a higher frequency of baseline deficits in attentional capacity, 8 (38.1%) versus 4 (10.3%), *p* = 0.003, respectively. Although this study proposed to record C-reactive protein (CRP) levels as a marker of inflammation at baseline, only 7 participants had a recorded level. The study also proposed to assess the ADS score as a potential baseline measure of participants’ anticholinergic activity profile, a potential risk factor for delirium, but due to interpretative difficulties this information was not recorded for any of the participants.

**Table 4:** Baseline risk factors according to occurrence of incident delirium

Characteristic	Study Group		All patients	***P*** value
	No Delirium ***n*** = 39 (%)	Delirium ***n*** = 21 (%)	Total ***n*** = 60 (%)	
**Age, yrs**^a^	67 (60–75)	68 (61–75)	67 (60–75)	0.687
**Sex: female**	20 (51.3)	7 (33.3)	27 (45.0)	0.183
**Primary cancer**				
**Lung**	13 (33.3)	7 (33.3)	20 (33.3)	0.544
**Gastrointestinal**	9 (23.1)	7 (33.3)	16 (26.7)	
**Genitourinary**	6 (15.4)	2 (9.5)	8 (13.3)	
**Breast**	5 (12.8)	1 (4.8)	6 (10.0)	
**Primary Brain**	1 (2.6)	1 (4.8)	2 (3.3)	
**Hematologic**	0 (0)	2 (9.5)	2 (3.3)	
**Head & Neck**	1 (2.6)	0 (0)	1 (1.7)	
**Other**	4 (10.3)	1 (4.8)	5 (8.3)	
**Metastatic sites of cancer**				
**Lungs**	14 (35.9)	6 (28.6)	20 (33.3)	0.566
**Liver**	17 (43.6)	9 (42.9)	26 (43.3)	0.956
**Brain**	11 (28.2)	9 (42.9)	20 (33.3)	0.251
**Leptomeninges**	3 (7.7)	3 (14.3)	6 (10.0)	0.078
**Bone**	21 (53.9)	10 (47.6)	31 (51.7)	0.645
**Charlson Comorbidity Index (CCI)**^a^	10 (9–12)	10 (9–13)	10 (9–12)	0.919
**Palliative Performance Score**^a^	40 (30–50)	40 (30–40)	40 (30–50)	**0.036**
**Baseline Goals of Care**				
**Comfort only**	7 (17.9)	6 (28.6)	13 (21.7)	0.301
**Medical**	23 (59.0)	14 (66.7)	37 (61.7)	
**Medical & Transfer to Acute Care**	8 (20.5)	1 (4.8)	9 (15.0)	
**Full Resuscitation**	1 (2.6)	0 (0)	1 (1.7)	
**Highest educational level achieved**				
**Elementary**	1 (2.6)	1 (4.76)	2 (3.3)	0.959
**Junior High School**	2 (5.13)	1 (4.76)	3 (5.00)	
**High School**	6 (15.4)	3 (14.3)	9 (15)	
**University / 3rd level**	12 (30.8)	8 (38.1)	20 (33.3)	
**Unknown**	18 (46.5)	8 (38.1)	26 (43.3)	
**Cognitive and psychiatric status at admission**				
**Short Orientation Memory Concentration Test Score (SOMCT)**^a^	2 (0–4)	6 (2–10)	2 (0–6)	**0.013**
**Recent neurocognitive symptoms (per admission history and assessments)**^b^				
**Perceptual disturbance**	3 (7.7)	3 (14.3)	6 (10.0)	0.417
**Delusional disturbance**	0 (0)	0 (0)	0 (0)	
**Attention problems**^**c**^	4 (10.3)	8 (38.1)	12 (20.0)	**0.003**
**Sleep pattern disturbed**^**c**^	7 (18.0)	3 (14.3)	10 (16.7)	0.443
**Insomnia severity index (ISI) score**^a^	10 (3–14)	5.5 (1–12.5)	9 (3–13)	0.336
**Previous delirium episode**				
**Documented occurrence**	7 (18.0)	6 (28.6)	13 (21.7)	0.562
**Unknown**	7 (18.0)	4 (19.1)	11 (18.3)	
**Dementia diagnosis**^**c**^	3 (7.7)	1 (4.8)	4 (6.7)	0.536
**Cognitive deficits without dementia**				
**Documented**	2 (5.1)	3 (14.3)	5 (8.3)	0.601
**Unknown**	2 (5.1)	1 (4.8)	3 (5.0)	
**Mood disorder diagnosis**^**c**^	7 (18.0)	1 (4.8)	8 (13.3)	0.268
**Anxiety disorder diagnosis**^**c**^	3 (7.7)	3 (14.3)	6 (10.0)	0.546
**Metabolic abnormalities at admission**				
**Hypoalbuminemia**	23 (59.0)	16 (76.2)	39 (65.0)	0.258
**Hyponatremia**	4 (10.3)	4 (19.1)	8 (13.3)	0.433
**Hypercalcemia**	2 (5.1)	1 (4.8)	3 (5.0)	1.000
**Low Hemoglobin (< 100 g/L)**	9 (23.1)	8 (38.1)	17 (28.3)	0.243
**Hypoxia requiring Oxygen therapy**	7 (18.0)	5 (23.8)	12 (20.0)	0.737
**Medications at admission**				
**Number of medications prescribed**^a^	12 (10–16)	10.5 (8–14)	12 (8–15)	0.187
**Psychoactive medications**^a^	2 (1–4)	2 (1–4)	2 (1–4)	0.641
**Morphine equivalent daily dose (MEDD)**^a^	30 (5–150)	60 (2.5–90)	54 (4.5–132)	0.944
**Diazepam equivalent daily dose**^a^	0 (0)	0 (0)	0 (0)	0.487
**Prednisone equivalent daily dose**^a^	12.25 (0–40)	13 (0–50)	12.5 (0–40)	0.868

^a^Continuous variables as median (Q1-Q3) unless otherwise reported; missing data in groups (delirium, no delirium) for some continuous variables: SOMCT score (3, 2); ISI score (0, 1); total number of medications prescribed (2, 1); psychoactive medications (2, 2); diazepam equivalent (4, 6); prednisone equivalent (1, 1)

^b^Features of subsyndromal delirium in the absence of a documented diagnosis of delirium

^c^Categorical variables for which there was missing data in groups (no delirium, delirium): highest educational level achieved (18, 8); dementia diagnosis documented (0, 1); cognitive impairment without dementia (2, 1); mood disorder (2, 0); anxiety disorder (5, 1); attention problems (2, 4); sleep disturbed (0, 1); missing data were counted as values in statistical analyses

**Fig. 3:**
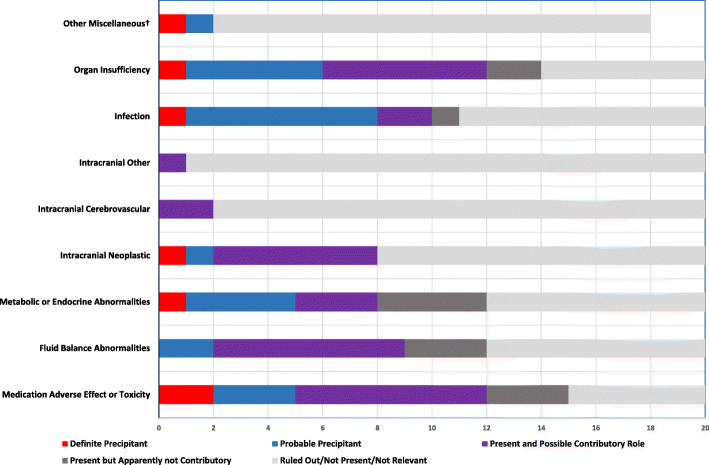
Categorized precipitants of delirium for 20 participants with incident delirium. †Data were not recorded in this category for two participants

Precipitant risk factors for incident delirium as identified through interview of the attending physicians, are presented in summarized category format both in Fig. 3 and Additional file 3, Table S3. There were no cases of medication or substance withdrawal, even in the “present but apparently not contributory” precipitant category. Infection, metabolic or endocrine abnormalities, organ insufficiency and medication adverse effect or toxicity ranked as the most frequent precipitants in the definite or probable categories.

#### Implementation of delirium assessment and management guidelines

The conduct of this feasibility study was used as an opportunity to implement standard clinical practice guidelines regarding delirium assessment and management on our PCU. These guidelines were implemented for all members of the inter-professional team in a modular fashion, with a focus on delirium screening, nonpharmacological interventions, and improving communication and support for patients and their families. Recommendations for antipsychotic prescribing were derived from recently published RCT evidence.

#### Safety evaluation

Although the total number of participants with at least one CTCAE Grade 3–5 serious adverse event was 17 (57%) in the placebo study group and 20 (67%) in the melatonin study group, these were considered to be not related (*n* = 15) or unlikely to be related (*n* = 2) in the placebo group and not related (*n* = 17) or unlikely to be related (*n* = 3) in the melatonin group.

## Discussion

There is a general consensus that a pilot or feasibility study is practically a prerequisite requirement prior to proceeding with a larger study, especially in palliative care settings, whose population are particularly vulnerable [56, 57]. Although the literature distinction between pilot and feasibility studies is somewhat unclear, [58, 59] there is some consensus that pilot studies are a subset of feasibility studies [60]. In meeting its primary and secondary objectives, the current study, although referred to as a feasibility study, provides useful information from both the pilot and feasibility perspectives prior to conducting the main RCT.

### Primary feasibility of study objectives

#### Recruitment and retention

The presence of delirium at inpatient admission in 31% of those not meeting inclusion criteria was the most frequent reason for study exclusion. Of the 616 screened subjects, 160 (26%) either declined to give informed consent or declined contact from the CRN/CRA. Approximately 10% of all screened subjects (*N* = 616) were recruited in this study and although there was no specific, a priori defined recruitment rate target, the recruitment at our single site required a lengthy period of approximately 16 months. From a recruitment perspective, these data indicate that a larger RCT would clearly require multisite collaboration.

Although a change in the study inclusion criteria to include participants with non-cancer end-of-life conditions might broaden the study target population and boost recruitment, it might also introduce a high degree of heterogeneity and add further challenges in terms of analyses. Nonetheless, this warrants consideration. The feasibility study recruited patients with advanced cancer that required inpatient PCU admission. It should be acknowledged that targeting patients earlier in their cancer disease trajectory might enhance recruitment potential for a community-based study (as opposed to an inpatient PCU study), but the validity of screening for and diagnosing delirium as an outcome in a community-based setting would also pose major challenges.

In terms of retention, 21 (35%) study participants contributed at-risk time from a time-to-event analysis perspective and exited the study prior to 21 days without developing delirium, thus requiring right censoring in the analysis. Of those who developed incident delirium, all but 2 did so by 21 days. These data suggest that we could consider shortening the duration of the main study to 21 days with minimal loss of data, which might be an acceptable trade-off, as multisite collaboration might be more attractive with a shorter study duration.

#### Preliminary data on outcome measures for the main study

Although the extent of missing assessment score data was substantial, this related mainly to outcomes that are designated as secondary outcome measures in the main study. The minimal protocol violation or deviation encountered in relation to the main study’s designated primary outcome measure supports its choice as primary outcome measure for the main study. Meanwhile, preliminary data obtained from the study regarding efficacy of study medications and sample size estimates warrant careful evaluation.

The study was remarkably informative regarding the time-to-event for the occurrence of incident delirium. However, caution should be exercised in relation to any hypothesis testing or interpreting efficacy differences between the study groups, as this study was not adequately powered to do so. Nonetheless, the signal highlights the importance of a DSMB and careful tracking of trends in both arms in the main study.

The study provided helpful pilot data in relation to the sample size requirement for the main study, originally estimated at 410. Although the 35% cumulative incidence of delirium in the study sample was higher than our pre-study projection of 25%, we under-estimated the need to censor cases in the time-to-event analysis: instead of the projected 10% withdrawal rate, there were 21 (35%) study participants who contributed at-risk time and required right censoring, albeit at varying times within the first 21 days of the study. Furthermore, our pre-study effect size change, reflected by a hazard ratio of 0.5 (a 50% reduction in hazard) and based on a previous study in a medical geriatric population, appears to be overly optimistic. Using a more conservative estimate for hazard ratio change to 0.7 (30% reduction in hazard), conducting a 21-day study and applying our feasibility/pilot data to our sample size estimation, together indicate a sample size requirement of 724 with 362 in each arm and 253 events (incident delirium) for the main study. This sample size requirement, 304 (72%) greater than the pre-study projection, highlights yet again the need for multisite collaboration in the main study, assuming that survival analysis with time-to-event (incident delirium diagnosis) is used as the primary outcome. An alternative approach, as adopted in a current Australian multi-site RCT designed to examine the role of melatonin in preventing delirium in PC settings, and in which members of our study team (MA, SB, PL, DC) are also involved, is to use delirium-free days as the primary outcome, while adjusting for length of inpatient stay. Using this as primary outcome has the advantage of having a smaller sample size requirement.

The severity of incident delirium (based on Nu-DESC, MDAS and CGR) and the severity of insomnia (based on ISI scores) are two secondary outcomes in the main study. The completion rates of Nu-DESC scores and ratings of CGR were acceptable and their respective scores were similar in the two study treatment groups, with the caveat that this study was not powered to detect a group difference. The proportions of missing MDAS scores (43%) and ISI scores for D14 (53%) and D28 (72%) precluded meaningful analyses in relation to both of these measures. The missing MDAS scores due to lack of CRN/CRA availability at weekends could be corrected by funding more extensive CRN/CRA coverage, whereas extreme patient fatigue and the consequent burden of conducting the MDAS assessment in such patients is less easily amenable to modification, albeit that based on available scores, pro-rating of scores for burdensome individual MDAS items has been reported [61]. Given that delirium severity measurement is a secondary rather than primary objective, consideration could be given to omitting the MDAS assessments in the main study for valid reasons: delirium severity is already captured by the CGR and the Nu-DESC, and our having previously reported moderate correlation between MDAS (as gold standard) and Nu-DESC scores in a Nu-DESC validation study [62]. The missing ISI scores due to patients’ discharge prior to reaching 28 days of the study could be addressed in the main study by omitting D14 and D21 ratings, and instead either conduct ratings on D7 of the study or rely solely on ESAS-r ratings for sleep.

#### Acceptability of study procedures and assessments

Acceptability of study procedures were reflected by a single protocol violation and a moderate degree of protocol deviations, mostly in relation to assessment tool ratings. Although CRN/CRA field notes indicated good acceptability of core study procedures from a patient and healthcare staff perspective, there were some remarkable instances of missing data, particularly in relation to the MDAS and ISI ratings, both amenable to solutions as previously discussed.

### Secondary objectives of the feasibility study

The study facilitated the implementation of delirium assessment and management guidelines, which will be reported separately. Safety evaluation of the study medication revealed no serious medication related adverse events. Although the discontinuation of melatonin in two participants with drowsiness was attributed as most likely due to other observed illness complications, we cannot exclude the potential for some contribution of melatonin to the drowsiness. The feasibility examination of collecting delirium risk factor data was highly informative towards planning a main study.

Although few studies have reported risk models for delirium in palliative care settings or palliative care eligible populations, [63] the baseline delirium risk factors identified in this study, poorer functional and cognitive performance and a higher frequency of baseline deficits in attentional capacity, are consistent with existing data from oncology settings [64, 65]. Data regarding CRP and ADS were not obtained as pre-planned. Although increasing anticholinergic drug burden in palliative care patients has been reported in association with delirium in a retrospective case-control study, [66] and cognitive impairment towards death in a secondary analysis of longitudinal data from an RCT, [67] there are remarkably conflicting data in many elderly and critical care studies regarding the role of anticholinergic drug burden, serum anticholinergic activity and scales to assess anticholinergic drug burden in relation to delirium risk [68–71]. In view of such uncertainty, anticholinergic drug burden evaluation could be omitted in the main study. In the case of low availability of CRP data, the goals of care on admission, designated as comfort only in 13 (22%) patients may have influenced physicians’ decision not to request CRP levels.

Moreover, the study demonstrated a temporal trend towards an increasing focus on comfort only: of the 21 participants who developed incident delirium, 12 (57%) had their goals of care designated as comfort only at that time. This trend is consistent with increasing awareness of disease progression and is in accordance with the fundamental principles of palliative care. However, the evaluation of predisposing and precipitating risk factors for delirium in a research study is compromised in this context, as confounding may occur due to the goals of care designation and its change over time. One ethically acceptable solution is to restrict investigation and evaluation of delirium risk factors to those whose goals of care are designated as “medical”, “medical and transfer” or “full resuscitation”.

In a larger RCT on delirium prevention, it is advisable to have good baseline data on covariates, pending patient consent for investigation, and adjust for them in the study analyses. More comprehensive epidemiological evaluation of delirium risk factors warrants a prospective cohort study to obtain robust data. The number of risk factors evaluated in the feasibility study was very large; it is possible that examination of existing databases may allow the selection of a smaller number of factors for either the planned RCT on prevention or a more comprehensive prospective cohort study of delirium risk factors [63].

### Strengths and limitations

This study met all of its clearly defined objectives and provided very useful feasibility and pilot data to inform the planning and conduct of a larger double blind RCT, albeit with some important modifications. The sample size of 60 was consistent with literature recommendations of at least 9% of the projected sample size of the main RCT and adequate to meet the objectives of the study. The study provided useful preliminary information to be cautiously interpreted in accordance with literature recommendations [72]. The feasibility study’s randomization process resulted in two study groups that were well balanced and comparable. The study had some important limitations apart from the limited ability to evaluate efficacy. First, although we did not survey patients regarding the effectiveness of blinding process, there were no signs of any deficits in this regard. Second, although the study identified substantive areas of missing data, particularly in relation to follow-up assessment tool scores and the recording of risk factors, this has enabled more appropriate planning towards a larger RCT. Overall, the distribution of missing baseline neurocognitive and other data across the main study groups was similar and likely of a random nature. Third, the potential for underdiagnosis of delirium among study participants at baseline cannot be excluded and could be reduced by gaining information from the addition of a more elaborate assessment tool such as the Delirium Rating-Revised-98 (DRS-98) [73] or MDAS at the baseline assessment.

## Conclusion

In meeting its pre-defined objectives, this study adequately assessed the feasibility of conducting a double blind RCT to examine the role of exogenous melatonin to prevent delirium in patients with advanced cancer who were admitted to an inpatient palliative care unit. A larger double-blind RCT is feasible, but subject accrual and withdrawal rates signal a need for adjustment in sample size and multisite collaboration. Although not powered for efficacy evaluation, the trend for shorter time to incident delirium in the melatonin group bodes for careful monitoring in a larger trial. The ethically acceptable evaluation of risk factors for delirium in research studies warrants careful consideration in light of the agreed goals of care and their temporal shift towards more comfort focussed care.

## Supplementary information

**Additional file 1 **: **Table S1**. Baseline Risk Profile for Delirium in the Cancer Trajectory (BRP-DICT)

**Additional file 2 **: **Table S2.** Precipitant Profile for Delirium in the Cancer Trajectory (PP-DICT)

**Additional file 3 **: **Table S3.** Aggregated frequency of delirium precipitants and their estimated role in incident delirium

## Data Availability

The datasets used and/or analysed during the current study are available from the corresponding author on reasonable request.

## Abbreviations

ADS: Anticholinergic drug scale
BRP-DICT: Baseline Risk Profile for Delirium in the Cancer Trajectory (BRP-DICT)
CAM: Confusion Assessment Method
CCI: Charlson Comorbidity Index
CGR: Clinician Global Rating
CI: Confidence interval
CONSORT: Consolidated Standards of Reporting Trials
CRA: Clinical research assistant
CRN: Clinical research nurse
CRP: C-reactive protein
DSMB: Data and Safety Monitoring Board
ECS-CP: Edmonton Classification System for Cancer Pain
ESAS-r: Edmonton Symptom Assessment System-revised
GOC: Goals of care
IC: Informed consent
ICH GCP: International Conference on Harmonization Good Clinical Practice
IR: Immediate release
ISI: Insomnia Severity Index
MDAS: Memorial Delirium Assessment Scale
NCI-CTCAE v4.03: National Cancer Institute Common Terminology Criteria for Adverse Events version4.03
Nu-DESC: Nursing Delirium Screening Scale
OHRI: Ottawa Hospital Research Institute
PC: Palliative care
PCU: Palliative care unit
PHIPA: Personal Health Information Protection Act
PP-DICT: Precipitant Profile for Delirium in the Cancer Trajectory
PPS: Palliative Performance Scale
RCT: Randomized controlled trial
SDM: Substitute decision-maker
SOMCT: Short Orientation-Memory-Concentration Test
TCPS 2: Tri-Council Policy Statement version 2
