# Supplementary material for: Melatonin to prevent delirium in patients with advanced cancer: a double blind, parallel, randomized, controlled, feasibility trial
Source: BMC Palliat Care. 2020 Oct 21;19:163. doi: 10.1186/s12904-020-00669-z (PMC7579814; doi:10.1186/s12904-020-00669-z)
Supplement: Supplementary file 1 — Additional file 1 : Table S1. Baseline Risk Profile for Delirium in the Cancer Trajectory (BRP-DICT) [file 12904_2020_669_MOESM1_ESM.docx]

**Additional file 1.**

**Table S1: Baseline Risk Profile for Delirium in the Cancer Trajectory (BRP-DICT)**

Please complete this at admission. Please X the boxes / circle Y (Yes), N (No) or UKN (Unknown) as appropriate.

| **1 Primary Cancer Diagnosis** |  |
| --- | --- |
| (a) Lung |  |
| (b) Breast |  |
| (c) Gastrointestinal |  |
| (d) Prostate |  |
| (d) Genitourinary excluding Prostate |  |
| (e) Hematological |  |
| (f) Primary Brain |  |
| (g) Head and Neck |  |
| (h) Other [ Specify: ] |  |
|  |  |
| **2 Presence of metastatic disease** |  |
| 1. Lung |  |
| 1. Hepatic |  |
| 1. Brain |  |
| 1. Leptomeningeal |  |
| 1. Bone |  |
| 1. Other [ Specify: ] |  |
|  |  |
| **3 Dementia diagnosis** |  |
| Documented diagnosis of dementia or collateral history consistent with the diagnosis of dementia | **Y / N / UKN** |
|  |  |
| **4 Previous recent episode of delirium** |  |
| Documented previous episode or collateral history consistent with an episode of delirium within the last year | **Y / N / UKN** |
|  |  |
| **4 Mood disorder** |  |
| Documented diagnosis of bipolar or depressive disorder or collateral history consistent with the diagnosis of bipolar or depressive disorder | **Y / N / UKN** |
|  |  |
| **5 Anxiety Disorder** |  |
| Documented diagnosis of anxiety disorder/ panic disorder/ obsessive compulsive disorder / phobias / post-traumatic stress disorder or other type of anxiety disorder or collateral history consistent with the diagnosis of depression | **Y / N / UKN** |
|  |  |
| **6 Maximum Educational Attainment Level** |  |
| 1. Elementary |  |
| 1. Junior High School |  |
| 1. High School |  |
| 1. University or Other 3rd Level |  |
| (e) Unknown |  |
|  |  |
| **7 Edmonton Classification System for Cancer Pain (ECS-CP) as completed on admission** |  |
| **Pain mechanism** |  |
| No Pain (No) |  |
| Nociceptive Pain (Nc) |  |
| Neuropathic or Mixed Pain (Ne) |  |
| Insufficient information to classify (Nx) |  |
|  |  |
| **Incident Pain** |  |
| No incident pain (Io) |  |
| Incident pain present (Ii) |  |
| Insufficient information to classify (Ix) |  |
|  |  |
| **Psychological Distress** |  |
| Psychological distress absent (Po) |  |
| Psychological distress present (Pp) |  |
| Insufficient information to classify (Px) |  |
|  |  |
| **Addiction History** |  |
| Addiction history absent (Ao) |  |
| Addiction history present (Aa) |  |
| Insufficient information to classify (Ax) |  |
|  |  |
| **8 Palliative Performance Scale Rating** |  |
| ≥ 70 % |  |
| 60 % |  |
| 50 % |  |
| 40 % |  |
| 30 % |  |
|  |  |
| **9 Cognitive impairment without dementia** |  |
| Short Orientation Memory Concentration Test (SOMCT) Score > 6 and <10 without a diagnosis of dementia | **Y / N / UKN** |
|  |  |
| **10 Specific Delirium Subsyndromal Features as per admission assessment** |  |
| (a) Perceptual disturbance: misperceptions or hallucinations | **Y / N / UKN** |
| (b) Delusional disturbance: paranoia, feeling unsafe | **Y / N / UKN** |
| (c) Attention deficit: ≥1 error on either counting 20 to 1 in reverse or stating the months backwards on SOMCT | **Y / N / UKN** |
| (d) Sleep-wake cycle disturbance: as per history or collateral history | **Y / N / UKN** |
|  |  |
| **11 Metabolic Status** |  |
| 1. Hypoalbuminaemia (at admission, using local reference range) |  |
| 1. Hyponatraemia (at admission, using local reference range) |  |
| 1. Hypercalcaemia (at admission or in the 28 days prior to   Admission, **corrected**: [40 – Albumin level] x [ calcium level]) |  |
| 1. Anaemia (Hb < 100 g/L at admission) |  |
| 1. Hypoxia, requiring oxygen to maintain saturations ≥ 90% |  |
|  |  |
| **12 Chronic inflammation index** |  |
| (a) C-Reactive Protein level (CRP in mg/L) | [ ] |
|  |  |
| **13 Medication Doses** |  |
| 1. Opioids in Total Morphine equivalent daily dose (oral) in mg | [ ] |
| 1. Corticosteroid daily dose in mg of Prednisone equivalent | [ ] |
| 1. Benzodiazepine diazepam daily equivalent in mg | [ ] |
| 1. Anticholinergic drug scale score | [ ] |
|  |  |
| **14 Polypharmacy Profile: Regular Medications, and PRN only medications (differing from regular medications) that were used at least once in the 48 hours preceding admission** |  |
| 1. Total number of medications prescribed | [ ] |
| (b) Total number of psychoactive medications prescribed | [ ] |
|  |  |
| **15 Comorbidity Measure** |  |
| (a) Charlson Comorbidity Index [0-31] | [ ] |
|  |  |
| **16 Admission Symptom Burden as per the Revised Version of the Edmonton Symptom Assessment System (ESAS-r) [0=best, 10=worst]** |  |
| 1. Pain | [ ] |
| 1. Tiredness | [ ] |
| 1. Drowsiness | [ ] |
| 1. Nausea | [ ] |
| 1. Lack of appetite | [ ] |
| 1. Shortness of breath | [ ] |
| 1. Depression | [ ] |
| 1. Anxiety | [ ] |
| 1. Well-being | [ ] |
| 1. Sleep | [ ] |
