# Supplementary material for: Melatonin to prevent delirium in patients with advanced cancer: a double blind, parallel, randomized, controlled, feasibility trial
Source: BMC Palliat Care. 2020 Oct 21;19:163. doi: 10.1186/s12904-020-00669-z (PMC7579814; doi:10.1186/s12904-020-00669-z)
Supplement: Supplementary file 2 — Additional file 2 : Table S2. Precipitant Profile for Delirium in the Cancer Trajectory (PP-DICT) [file 12904_2020_669_MOESM2_ESM.docx]

**Additional file 2**

**Table S2: Precipitant Profile for Delirium in the Cancer Trajectory (PP-DICT)**

This checklist profile of delirium precipitants and their collective categories includes most of the commonly encountered ones in the cancer disease trajectory. An individual patient precipitant profile is created by using a weighted approach for documenting the range of potential inputs in any single episode of delirium. Therefore, raters may indicate multiple categories as contributing toward reaching the threshold for delirium. The relative importance of history, examination, and tests in supporting the significance of any particular precipitating factor will vary among cases and episodes. Consequently, the certainty of a precipitant role will depend on the availability of information and the judgement of the clinician involved. Specific precipitants are assigned to categories as presented below. **Please “X” a box for each row as appropriate.**

|  | Definite Precipitant | Probable Precipitant | Present and Possible Contributory Role | Present but Apparently not Contributory | Ruled Out/Not Present/Not Relevant |
| --- | --- | --- | --- | --- | --- |
|  | 4 | 3 | 2 | 1 | 0 |
| 1 Medication Adverse Effect or  Toxicity |  |  |  |  |  |
| 2 Medication or Substance  Withdrawal |  |  |  |  |  |
| 3 Fluid Balance Abnormalities |  |  |  |  |  |
| 4 Metabolic or Endocrine  Abnormalities |  |  |  |  |  |
| 5 Intracranial Neoplastic |  |  |  |  |  |
| 6 Intracranial Cerebrovascular |  |  |  |  |  |
| 7 Intracranial Other |  |  |  |  |  |
| 8 Infection |  |  |  |  |  |
| 9 Organ Insufficiency |  |  |  |  |  |
| 10 Other Miscellaneous |  |  |  |  |  |

- On pages 2-5, a detailed list of recognized potential precipitants is grouped under each of the above categories. Please **identify the precipitant [ ]** where appropriate and **rate each one (X)** in terms of their contributory role (definite, probable, or possible etc) in the episode of delirium.
- Note the **“definite”** category designation implies a clear, indisputable temporal association with the episode of delirium, absence of any other “possible” or “probable” precipitants, and recognition as a precipitant of delirium.
- The **“probable”** designation implies that it is the sole precipitant in the “probable” category, has an indisputably clear temporal association with the delirium episode and ≥1 “possible” precipitants are also present.
- A **“possible”** designation refers to situations where there are one or more potential precipitants present but the availability of information or the temporal association with the delirium is less evident than with the “definite” or “probable” categories.

| **Precipitant Profile for Delirium in the Cancer Trajectory (PP-DICT)** | | | | | |
| --- | --- | --- | --- | --- | --- |
| **Collective Precipitant Category and Precipitants**  **Please specify where appropriate [ ]** | **Definite Precipitant** | **Probable Precipitant** | **Present and Possible Contributory Role** | **Present but Apparently not Contributory** | **Ruled Out/Not Present/Not Relevant** |
|  | **4** | **3** | **2** | **1** | **0** |
| **1 Medication Adverse Effect or Toxicity** |  |  |  |  |  |
| (a) Tricyclic antidepressant (TCA) |  |  |  |  |  |
| (b) Selective Serotonin Reuptake Inhibitor (SSRI) |  |  |  |  |  |
| (c) SSN(Noradrenalin) RI (SSNRI) |  |  |  |  |  |
| (d) Psychostimulant |  |  |  |  |  |
| (e) Benzodiazepine or other sedatives |  |  |  |  |  |
| (f) Lithium |  |  |  |  |  |
| (g) Neuroleptic / Antipsychotic |  |  |  |  |  |
| (h) Corticosteroid |  |  |  |  |  |
| (i) Ketamine |  |  |  |  |  |
| (j) Antiepileptic / Anticonvulsant |  |  |  |  |  |
| (k) Anticholinergic with central action |  |  |  |  |  |
| (l) Opioid analgesic [Specify: ] |  |  |  |  |  |
| (m) Antihistamine |  |  |  |  |  |
| (n) Other psychotropic medication [ ] |  |  |  |  |  |
| (o) Other non-psychotropic medication [ ] |  |  |  |  |  |
|  |  |  |  |  |  |
|  |  |  |  |  |  |
| **2 Medication or Substance Withdrawal** |  |  |  |  |  |
| (a) Benzodiazepine |  |  |  |  |  |
| (b) Opioid |  |  |  |  |  |
| (c) Alcohol |  |  |  |  |  |
| (d) Other [ Specify: ] |  |  |  |  |  |

| **Collective Precipitant Category and Precipitants**  **Please specify where appropriate [ ]** | **Definite Precipitant** | **Probable Precipitant** | **Present and Possible Contributory Role** | **Present but Apparently not Contributory** | **Ruled Out/Not Present/Not Relevant** |
| --- | --- | --- | --- | --- | --- |
|  | **4** | **3** | **2** | **1** | **0** |
| **3 Fluid Balance Abnormalities** |  |  |  |  |  |
| (a) Hyponatraemia |  |  |  |  |  |
| (b) Clinically dehydrated |  |  |  |  |  |
| (c) Clinically hypovolemic due to presence of ≥ 1 of following: [blood loss], [vomiting], [diarrhoea], [fistula loss], or 3rd-spacing of fluid due to [oedema], [ascites] or [pleural effusion] |  |  |  |  |  |
| (d) Laboratory evidence: Hypernatraemia |  |  |  |  |  |
| (e) Laboratory evidence: Elevated urea with normal creatinine in the absence of gastrointestinal bleeding |  |  |  |  |  |
| (f) Other [ Specify: ] |  |  |  |  |  |
|  |  |  |  |  |  |
| **4 Metabolic or Endocrine** **Abnormalities** |  |  |  |  |  |
| (a) Hypoxia (Oxygen saturation < 90%) |  |  |  |  |  |
| (b) Anaemia (Haemoglobin < 100 g/L) |  |  |  |  |  |
| (c) Hypoglycaemia |  |  |  |  |  |
| (d) Hypercalcaemia (corrected) |  |  |  |  |  |
| (e) Hypomagnesemia |  |  |  |  |  |
| (f) Hypothyroidism |  |  |  |  |  |
| (g) Hyperthyroidism |  |  |  |  |  |
| (h) Other [Specify: ] |  |  |  |  |  |
|  |  |  |  |  |  |
|  |  |  |  |  |  |
|  |  |  |  |  |  |

| **Collective Precipitant Category and Precipitants**  **Please specify where appropriate [ ]** | **Definite Precipitant** | **Probable Precipitant** | **Present and Possible Contributory Role** | **Present but Apparently not Contributory** | **Ruled Out/Not Present/Not Relevant** |
| --- | --- | --- | --- | --- | --- |
|  | **4** | **3** | **2** | **1** | **0** |
| **5 Intracranial Neoplastic** |  |  |  |  |  |
| (a) Primary brain tumour |  |  |  |  |  |
| (b) Metastatic brain disease |  |  |  |  |  |
| (c) Leptomeningeal metastatic disease |  |  |  |  |  |
| (d) Other [ Specify: ] |  |  |  |  |  |
|  |  |  |  |  |  |
| **6 Intracranial Cerebrovascular** |  |  |  |  |  |
| (a) Transient Ischaemic Attack (TIA) |  |  |  |  |  |
| (b) Cerebrovascular Accident (CVA) |  |  |  |  |  |
| (c) Subdural Haematoma |  |  |  |  |  |
| (d) Other [ Specify: ] |  |  |  |  |  |
|  |  |  |  |  |  |
| **7 Intracranial Other** |  |  |  |  |  |
| (a) Seizure |  |  |  |  |  |
| (b) Head Injury / Traumatic Brain Injury |  |  |  |  |  |
| (c) Other [ Specify: ] |  |  |  |  |  |
|  |  |  |  |  |  |
| **8 Infection** |  |  |  |  |  |
| (a) Bacterial Urinary Tract Infection |  |  |  |  |  |
| (b) Clinical Evidence: Lower Respiratory Tract Infection (Bacterial or Nonbacterial) |  |  |  |  |  |
| (c) Bacterial Skin / Wound Infection |  |  |  |  |  |
| (d) Other Bacterial such as [Peritonitis] [ ] |  |  |  |  |  |
| (e) Nonbacterial infection [ ] |  |  |  |  |  |
|  |  |  |  |  |  |
|  |  |  |  |  |  |

| **Collective Precipitant Category and Precipitants**  **Please specify where appropriate [ ]** | **Definite Precipitant** | **Probable Precipitant** | **Present and Possible Contributory Role** | **Present but Apparently not Contributory** | **Ruled Out/Not Present/Not Relevant** |
| --- | --- | --- | --- | --- | --- |
|  | **4** | **3** | **2** | **1** | **0** |
| **9 Organ Insufficiency^§^** |  |  |  |  |  |
| (a) Cardiac |  |  |  |  |  |
| (b) Pulmonary |  |  |  |  |  |
| (c) Hepatic |  |  |  |  |  |
| (d) Renal |  |  |  |  |  |
| (e) Adrenal |  |  |  |  |  |
| (f) Bone marrow |  |  |  |  |  |
| (g) Other [ Specify: ] |  |  |  |  |  |
|  |  |  |  |  |  |
|  |  |  |  |  |  |
| **10 Other Miscellaneous Category** |  |  |  |  |  |
| (a) Room change within 2 days prior to delirium diagnosis |  |  |  |  |  |
| (b) Number of room changes since admission |  |  |  |  |  |
| (c) Other [ Specify: ] |  |  |  |  |  |
|  |  |  |  |  |  |
|  |  |  |  |  |  |

§ Based on attending physicians’ assessment of clinical findings, laboratory results and diagnostic imaging studies.
