# Supplementary material for: Melatonin to prevent delirium in patients with advanced cancer: a double blind, parallel, randomized, controlled, feasibility trial
Source: BMC Palliat Care. 2020 Oct 21;19:163. doi: 10.1186/s12904-020-00669-z (PMC7579814; doi:10.1186/s12904-020-00669-z)
Supplement: Supplementary file 3 — Additional file 3 : Table S3. Aggregated frequency of delirium precipitants and their estimated role in incident delirium [file 12904_2020_669_MOESM3_ESM.docx]

**Additional file 3**

**Table S3: Aggregated frequency of delirium precipitants and their estimated role in incident delirium***

|  | Definite Precipitant | Probable Precipitant | Present and Possible Contributory Role | Present but Apparently not Contributory | Ruled Out/Not Present/Not Relevant |
| --- | --- | --- | --- | --- | --- |
| Medication Adverse Effect or Toxicity | 2 | 3 | 7 | 3 | 5 |
| Medication or Substance Withdrawal | 0 | 0 | 0 | 0 | 20 |
| Fluid Balance Abnormalities | 0 | 2 | 7 | 3 | 8 |
| Metabolic or Endocrine Abnormalities | 1 | 4 | 3 | 4 | 8 |
| Intracranial Neoplastic | 1 | 1 | 6 | 0 | 12 |
| Intracranial Cerebrovascular | 0 | 0 | 2 | 0 | 18 |
| Intracranial Other | 0 | 0 | 1 | 0 | 19 |
| Infection | 1 | 7 | 2 | 1 | 9 |
| Organ Insufficiency | 1 | 5 | 6 | 2 | 6 |
| Other Miscellaneous† | 1 | 1 | 0 | 0 | 16 |

*Estimates for 20 participants with incident delirium; data were not recorded for one participant.

†Data for two participants were not recorded
